# *catena*-Poly[[chlorido­tris­(1,3-thia­zolidine-2-thione-κ*S*)cadmium(II)]-μ-chlorido]

**DOI:** 10.1107/S2414314620014236

**Published:** 2020-10-30

**Authors:** Aboubacar Diop, Tidiane Diop, Antoine Blaise Kama, Cheikh Abdou Khadre Diop, Nikolay Tumanov

**Affiliations:** a Laboratoire de Chimie Minérale et Analytique (LA.CHI.MI.A), Département de Chimie, Faculté des Sciences et Techniques, Université Cheikh Anta Diop, Dakar, Senegal; b Departement de Chimie, Université de Namur, Rue de Bruxelles 61-5000, Namur, Belgium; Goethe-Universität Frankfurt, Germany

**Keywords:** crystal structure, 1,3-thia­zolidine-2-thione, cadmium centre, hydrogen bonding

## Abstract

The synthesis and characterization of poly[di­chlorido­tri(1,3-thia­zolidine-2-thione)cadmium(II)], [CdCl_2_(C_3_H_5_NS_2_)_3_]_*n*_, prepared from CdCl_2_·H_2_O and C_3_H_5_NS_2_ (tzdSH) in a 1:3 ratio, are described. The Cd^II^ cation is coordinated by three 1,3-thia­zolidine-2-thione mol­ecules and three Cl^−^ anions in a distorted octa­hedral environment. The Cd metal centres are connected *via* Cl^−^ ligands, creating polymeric chains running along the *a-*axis direction. The conformation of the chains is stabilized by N—H⋯Cl hydrogen bonds.

## Abstract 2

In the structure of the title compound, [CdCl_2_(C_3_H_5_NS_2_)_3_]_n_, the Cd^II^ atom is coordinated by three S and three Cl atoms in a *mer* arrangement.

## Graphical abstract

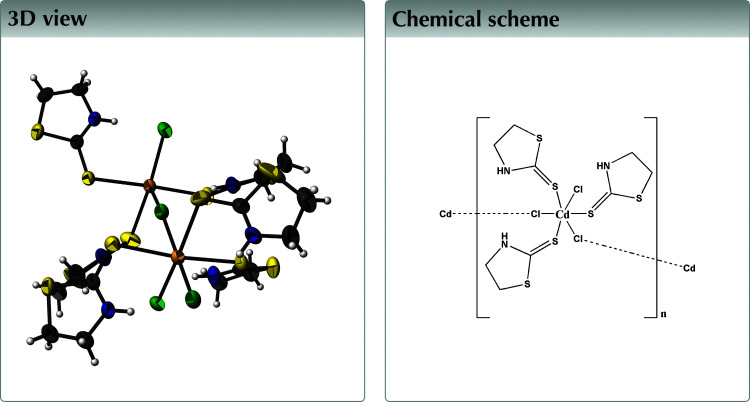

## Structure description

1,3-Thia­zolidine-2-thione (tzdSH: C_3_H_5_NS_2_), is a well-known heterocyclic thione/thiol ligand. Crystallographic studies and investigations of its modes of coordination have been reported (Saithong *et al.*, 2007 ▸). We are inter­ested in the coordination behaviour and structure of tzdSH complexes with Cd^II^ chloride. The synthesis is accompanied by a transformation of the tzdSH (tzdSH: C_3_H_5_NS_2_, thiol form) into a tzdt ligand (tzdt: C_3_H_5_NS_2_, thion form). A similar transformation was described previously by Saithong *et al.* (2014 ▸). Metal complexes of thio­nes and thio­nates were reviewed by Raper (1997 ▸).

Cadmium (II) is known to form a wide variety of 1:1 to 1:4 complexes with thio­nes, where the structural arrangements are generally tetra­hedral and octa­hedral coordination environments (Mahmood *et al.*, 2018 ▸). The 1:1 complexes, for example [Cd(Melmt)(S_e_CN)] (Melmt = *N*-methyl­imidazolidine-2-thione; Fettouhi *et al.*, 2008 ▸) usually exist in the polymeric form. The 1:2 complexes such as [Cd(Dmtu)_2_ *X*_2_] (Dmtu = *N*,*N*′-di­methyl­thio­urea-κ*S*; *X* = Cl, Br, I; Ahmad *et al.*, 2011 ▸) are the most common and often consist of discrete monomeric mol­ecules with a terahedrally (Moloto *et al.*, 2003 ▸) or octa­hedrally (Mahmood *et al.*, 2012 ▸) coordinated Cd^II^ ion. The 1:3 compounds are rare: the structure of [Cd(Tu)_3_(SO_4_)] shows that the complex is a dimer, and the coordination around the metal atom is inter­mediate between square pyramidal and trigonal bipyramidal (Corao & Baggio, 1969 ▸). The 1:4 complexes may be ionic or non-ionic (Mahmood *et al.*, 2018 ▸).

**Figure 1:**
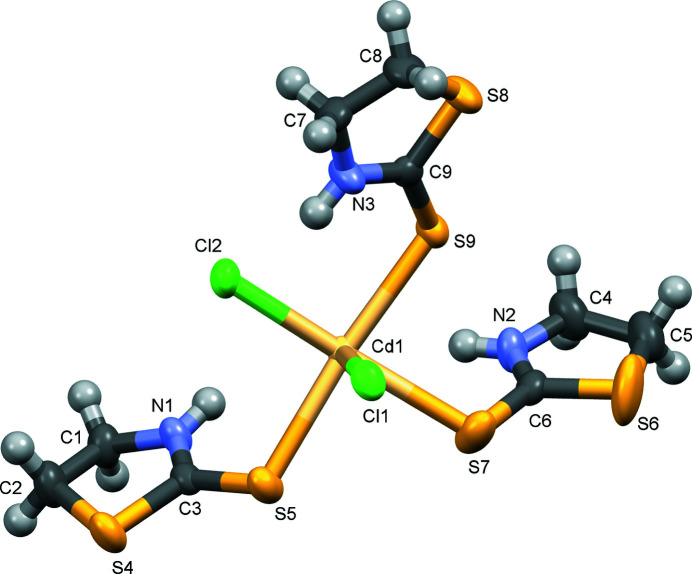
The asymmetric unit of title compound with displacement ellipsoids drawn at the 50% probability level.

The above structural studies show that thio­nes coordinate to cadmium (II) *via* the sulfur atom. To further investigate the structural aspects of such complexes, we report in this work a complex with a Cd^II^:thione ratio of 1:23. The asymmetric unit consists of a cadmium (II) ion bonded to three 1,3-thia­zolidine-2-thione moieties *via* the exocyclic sulfur atom and two Cl atoms (Fig. 1 ▸). The Cd—S and Cd—Cl bond lengths are in the range 2.7004 (11)–2.7347 (13) and 2.5430 (12)–2.7258 (16) Å, respectively. The bond lengths are slightly different from those reported in the literature [Cd—S = 2.604 Å and Cd—Cl = 2.7105 Å; Bell *et al.*, 2004 ▸]. This may be due to the intra­molecular hydrogen bonds observed in the crystal structure.

**Figure 2:**
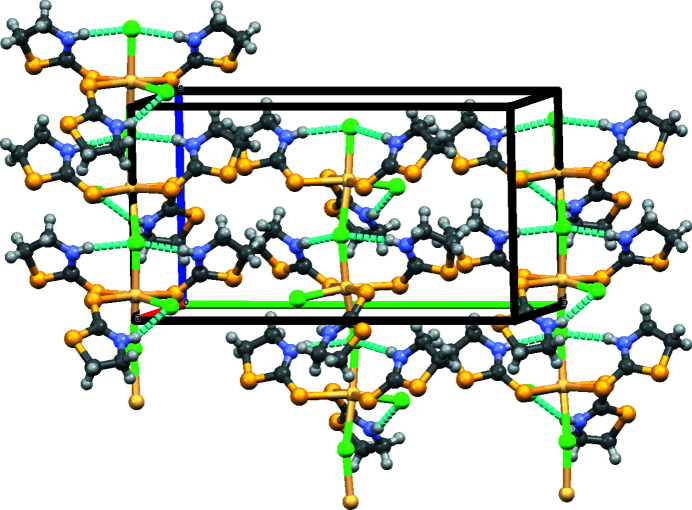
Packing diagram of the title compound. N—H⋯Cl hydrogen bonds are show as light blue dashed lines.

In the crystal, one of Cl^−^ anions connects two neighbouring Cd^II^ centers leading to polymeric chains. No hydrogen bonds are observed between the chains. The structure of the compound can be described as parallel chains running along the *a-*axis direction. The conformation of the chains is stabilized by N—H⋯Cl hydrogen bonds (Table 1 ▸, Fig. 2 ▸).

## Synthesis and crystallization

In a round-bottom flask, to the ligand (tzdSH: C_3_H_5_NS_2_) (15 mmol, 1.79 g) in 5 mL of 1,4-dioxan, a solution of CdCl_2_·H_2_O (5 mmol, 1.01 g) in 5 mL of distilled water was added. The mixture was refluxed for 4 h. Light-yellow crystals appeared after the light yellow filtrate had been kept at room temperature for two days (yield 75%).

## Refinement

Crystal data, data collection and structure refinement details are summarized in Table 2 ▸.

## Supplementary Material

Crystal structure: contains datablock(s) I. DOI: 10.1107/S2414314620014236/bt4100sup1.cif

Structure factors: contains datablock(s) I. DOI: 10.1107/S2414314620014236/bt4100Isup2.hkl

CCDC reference: 2040604

Additional supporting information:  crystallographic information; 3D view; checkCIF report

## Figures and Tables

**Table 1:** Hydrogen-bond geometry (Å, °)

*D*—H⋯*A*	*D*—H	H⋯*A*	*D*⋯*A*	*D*—H⋯*A*
N1—H1⋯Cl1^i^	0.86	2.43	3.260 (4)	163
N2—H2⋯Cl1^i^	0.86	2.47	3.314 (4)	168
N3—H3⋯Cl2	0.86	2.41	3.165 (4)	147
C8—H8*B*⋯Cl1^ii^	0.97	2.82	3.781 (6)	171

Symmetry codes: (i) 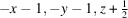 ; (ii) 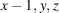 .

**Table 2:** Experimental details

Crystal data	
Chemical formula	[CdCl_2_(C_3_H_5_NS_2_)_3_]
*M*_r_	540.90
Crystal system, space group	Orthorhombic, *P* *n* *a*2_1_
Temperature (K)	295
*a*, *b*, *c* (Å)	9.2014 (3), 19.3472 (6), 10.5827 (3)
*V* (Å^3^)	1883.94 (9)
*Z*	4
Radiation type	Mo *K*α
μ (mm^−1^)	2.10
Crystal size (mm)	0.76 × 0.50 × 0.14

Data collection	
Diffractometer	Oxford Diffraction Xcalibur, Ruby, Gemini Ultra
Absorption correction	Gaussian (*CrysAlis PRO*; Rigaku OD, 2018 ▸)
*T*_min_, *T*_max_	0.242, 1.000
No. of measured, independent and observed [*I* > 2σ(*I*)] reflections	12033, 5594, 4781
*R*_int_	0.026
(sin θ/λ)_max_ (Å^−1^)	0.714

Refinement	
*R*[*F* ^2^ > 2σ(*F* ^2^)], *wR*(*F* ^2^), *S*	0.033, 0.069, 1.01
No. of reflections	5594
No. of parameters	190
No. of restraints	1
H-atom treatment	H-atom parameters constrained
Δρ_max_, Δρ_min_ (e Å^−3^)	0.40, −0.79
Absolute structure	Flack *x* determined using 1872 quotients [(*I* ^+^)−(*I* ^−^)]/[(*I* ^+^)+(*I* ^−^)] (Parsons *et al.*, 2013 ▸)
Absolute structure parameter	−0.010 (19)

Computer programs: *CrysAlis PRO* (Rigaku OD, 2018 ▸), *SHELXT* (Sheldrick, 2015*a* ▸) and *SHELXL2018/3* (Sheldrick, 2015*b* ▸).

## full crystallographic data

### Crystal data

**Table:**

[CdCl_2_(C_3_H_5_NS_2_)_3_]	*D*_x_ = 1.907 Mg m^−^^3^
*M**_r_* = 540.90	Mo *K*α radiation, λ = 0.71073 Å
Orthorhombic, *P**n**a*2_1_	Cell parameters from 4396 reflections
*a* = 9.2014 (3) Å	θ = 3.1–32.5°
*b* = 19.3472 (6) Å	µ = 2.10 mm^−^^1^
*c* = 10.5827 (3) Å	*T* = 295 K
*V* = 1883.94 (9) Å^3^	Block, colourless
*Z* = 4	0.76 × 0.50 × 0.14 mm
*F*(000) = 1072	

### Data collection

**Table:**

Oxford Diffraction Xcalibur, Ruby, Gemini Ultra diffractometer	5594 independent reflections
Graphite monochromator	4781 reflections with *I* > 2σ(*I*)
Detector resolution: 10.3712 pixels mm^-1^	*R*_int_ = 0.026
ω scans	θ_max_ = 30.5°, θ_min_ = 2.1°
Absorption correction: gaussian (CrysAlisPro; Rigaku OD, 2018)	*h* = −12→13
*T*_min_ = 0.242, *T*_max_ = 1.000	*k* = −27→27
12033 measured reflections	*l* = −14→15

### Refinement

**Table:**

Refinement on *F*^2^	Secondary atom site location: dual
Least-squares matrix: full	Hydrogen site location: mixed
*R*[*F*^2^ > 2σ(*F*^2^)] = 0.033	H-atom parameters constrained
*wR*(*F*^2^) = 0.069	*w* = 1/[σ^2^(*F*_o_^2^) + (0.0275*P*)^2^] where *P* = (*F*_o_^2^ + 2*F*_c_^2^)/3
*S* = 1.01	(Δ/σ)_max_ = 0.001
5594 reflections	Δρ_max_ = 0.40 e Å^−^^3^
190 parameters	Δρ_min_ = −0.78 e Å^−^^3^
1 restraint	Absolute structure: Flack *x* determined using 1872 quotients [(*I*^+^)-(*I*^-^)]/[(*I*^+^)+(*I*^-^)] (Parsons *et al.*, 2013)
Primary atom site location: dual	Absolute structure parameter: −0.010 (19)

### Special details

**Table:**

Geometry. All esds (except the esd in the dihedral angle between two l.s. planes) are estimated using the full covariance matrix. The cell esds are taken into account individually in the estimation of esds in distances, angles and torsion angles; correlations between esds in cell parameters are only used when they are defined by crystal symmetry. An approximate (isotropic) treatment of cell esds is used for estimating esds involving l.s. planes.
Refinement. H atoms were refined using a riding model with N—H = 0.86 Å or C—H = 0.97 Å and U(H)=1.2*U*_eq_(C,*N*).

### Fractional atomic coordinates and isotropic or equivalent isotropic displacement parameters (Å^2^)

**Table:**

	*x*	*y*	*z*	*U*_iso_*/*U*_eq_	
Cd1	−0.51629 (3)	−0.49641 (2)	−0.38259 (5)	0.03293 (9)	
Cl1	−0.46306 (13)	−0.51075 (6)	−0.63461 (14)	0.0402 (3)	
Cl2	−0.66097 (13)	−0.38657 (6)	−0.42258 (12)	0.0459 (3)	
S4	−0.17107 (15)	−0.28169 (7)	−0.32587 (13)	0.0514 (3)	
S5	−0.26212 (12)	−0.42407 (7)	−0.39534 (14)	0.0470 (3)	
S6	−0.4464 (3)	−0.76006 (9)	−0.31381 (18)	0.0922 (7)	
S7	−0.36450 (15)	−0.61793 (7)	−0.38167 (16)	0.0594 (4)	
S8	−1.01474 (16)	−0.59987 (9)	−0.54807 (18)	0.0592 (4)	
S9	−0.75414 (11)	−0.57811 (6)	−0.38890 (14)	0.0401 (2)	
N1	−0.3576 (4)	−0.3483 (2)	−0.1994 (3)	0.0415 (9)	
H1	−0.420149	−0.379411	−0.179291	0.050*	
N2	−0.5236 (5)	−0.6583 (2)	−0.1835 (4)	0.0483 (11)	
H2	−0.532251	−0.616145	−0.158878	0.058*	
N3	−0.8589 (5)	−0.4936 (2)	−0.5693 (4)	0.0515 (12)	
H3	−0.786934	−0.465732	−0.559655	0.062*	
C1	−0.3397 (6)	−0.2853 (3)	−0.1234 (5)	0.0504 (13)	
H1A	−0.282523	−0.295177	−0.048554	0.061*	
H1B	−0.433815	−0.267960	−0.097001	0.061*	
C2	−0.2647 (6)	−0.2334 (3)	−0.2030 (5)	0.0574 (14)	
H2A	−0.195748	−0.207054	−0.153008	0.069*	
H2B	−0.334355	−0.201629	−0.239964	0.069*	
C3	−0.2750 (5)	−0.3550 (2)	−0.2982 (4)	0.0352 (10)	
C4	−0.5903 (8)	−0.7138 (3)	−0.1123 (6)	0.0678 (18)	
H4A	−0.695161	−0.708523	−0.112475	0.081*	
H4B	−0.556857	−0.712710	−0.025382	0.081*	
C5	−0.5487 (8)	−0.7815 (3)	−0.1731 (6)	0.075 (2)	
H5A	−0.634932	−0.807737	−0.194980	0.090*	
H5B	−0.489916	−0.808860	−0.115812	0.090*	
C6	−0.4508 (5)	−0.6730 (3)	−0.2845 (4)	0.0403 (11)	
C7	−0.9747 (5)	−0.4783 (3)	−0.6584 (6)	0.0509 (14)	
H7A	−0.934127	−0.462911	−0.738207	0.061*	
H7B	−1.036634	−0.441940	−0.625452	0.061*	
C8	−1.0607 (6)	−0.5430 (3)	−0.6772 (6)	0.0596 (16)	
H8A	−1.035978	−0.564345	−0.757395	0.072*	
H8B	−1.163928	−0.532937	−0.676816	0.072*	
C9	−0.8670 (5)	−0.5509 (3)	−0.5041 (4)	0.0356 (10)	

### Atomic displacement parameters (Å^2^)

**Table:**

	*U*^11^	*U*^22^	*U*^33^	*U*^12^	*U*^13^	*U*^23^
Cd1	0.04166 (16)	0.03310 (15)	0.02402 (13)	−0.00411 (12)	0.0009 (2)	−0.00111 (16)
Cl1	0.0570 (9)	0.0418 (7)	0.0218 (4)	−0.0097 (5)	0.0013 (7)	−0.0008 (6)
Cl2	0.0537 (6)	0.0311 (6)	0.0529 (7)	0.0001 (5)	0.0046 (5)	−0.0039 (5)
S4	0.0563 (7)	0.0483 (8)	0.0496 (6)	−0.0205 (7)	0.0097 (6)	−0.0042 (7)
S5	0.0468 (6)	0.0463 (7)	0.0478 (7)	−0.0101 (5)	0.0080 (6)	−0.0116 (7)
S6	0.175 (2)	0.0366 (8)	0.0653 (9)	0.0215 (12)	0.0102 (13)	−0.0082 (8)
S7	0.0625 (7)	0.0549 (8)	0.0609 (8)	0.0190 (7)	0.0232 (9)	0.0123 (9)
S8	0.0489 (8)	0.0566 (10)	0.0720 (9)	−0.0200 (7)	−0.0169 (7)	0.0102 (8)
S9	0.0440 (5)	0.0367 (5)	0.0396 (5)	−0.0068 (4)	−0.0045 (6)	0.0030 (7)
N1	0.049 (2)	0.037 (2)	0.038 (2)	−0.012 (2)	0.0054 (18)	−0.0005 (18)
N2	0.070 (3)	0.034 (2)	0.041 (2)	−0.005 (2)	0.006 (2)	−0.0051 (19)
N3	0.048 (3)	0.045 (3)	0.062 (3)	−0.014 (2)	−0.018 (2)	0.011 (2)
C1	0.068 (3)	0.039 (3)	0.044 (3)	−0.002 (3)	0.003 (3)	−0.003 (3)
C2	0.075 (4)	0.042 (3)	0.056 (3)	−0.011 (3)	0.007 (3)	−0.010 (3)
C3	0.037 (2)	0.036 (2)	0.033 (2)	−0.005 (2)	−0.0081 (18)	0.0030 (19)
C4	0.085 (5)	0.058 (4)	0.060 (4)	−0.021 (4)	0.005 (3)	0.006 (3)
C5	0.092 (5)	0.047 (4)	0.087 (5)	−0.011 (4)	−0.014 (4)	0.010 (4)
C6	0.044 (2)	0.036 (3)	0.041 (3)	0.007 (2)	−0.008 (2)	−0.001 (2)
C7	0.047 (3)	0.049 (3)	0.057 (4)	0.009 (3)	−0.013 (3)	0.005 (3)
C8	0.044 (3)	0.064 (4)	0.071 (4)	−0.003 (3)	−0.021 (3)	0.002 (3)
C9	0.034 (2)	0.037 (3)	0.036 (2)	0.001 (2)	0.0045 (17)	−0.0071 (19)

### Geometric parameters (Å, º)

**Table:**

Cd1—Cl2	2.5430 (12)	N2—H2	0.8600
Cd1—Cl1^i^	2.6348 (16)	N3—C9	1.307 (6)
Cd1—S9	2.7004 (11)	N3—C7	1.454 (7)
Cd1—Cl1	2.7258 (16)	N3—H3	0.8600
Cd1—S5	2.7289 (12)	C1—C2	1.482 (7)
Cd1—S7	2.7347 (13)	C1—H1A	0.9700
S4—C3	1.736 (5)	C1—H1B	0.9700
S4—C2	1.818 (5)	C2—H2A	0.9700
S5—C3	1.690 (5)	C2—H2B	0.9700
S6—C6	1.714 (5)	C4—C5	1.509 (9)
S6—C5	1.810 (7)	C4—H4A	0.9700
S7—C6	1.679 (5)	C4—H4B	0.9700
S8—C9	1.721 (5)	C5—H5A	0.9700
S8—C8	1.804 (6)	C5—H5B	0.9700
S9—C9	1.686 (5)	C7—C8	1.495 (9)
N1—C3	1.299 (5)	C7—H7A	0.9700
N1—C1	1.469 (6)	C7—H7B	0.9700
N1—H1	0.8600	C8—H8A	0.9700
N2—C6	1.293 (6)	C8—H8B	0.9700
N2—C4	1.448 (7)		
			
Cl2—Cd1—Cl1^i^	94.82 (4)	C1—C2—H2A	110.5
Cl2—Cd1—S9	93.48 (4)	S4—C2—H2A	110.5
Cl1^i^—Cd1—S9	89.83 (4)	C1—C2—H2B	110.5
Cl2—Cd1—Cl1	90.94 (4)	S4—C2—H2B	110.5
Cl1^i^—Cd1—Cl1	173.143 (17)	H2A—C2—H2B	108.7
S9—Cd1—Cl1	93.55 (4)	N1—C3—S5	127.6 (4)
Cl2—Cd1—S5	90.68 (4)	N1—C3—S4	112.1 (4)
Cl1^i^—Cd1—S5	94.82 (4)	S5—C3—S4	120.3 (3)
S9—Cd1—S5	173.48 (5)	N2—C4—C5	108.3 (5)
Cl1—Cd1—S5	81.36 (4)	N2—C4—H4A	110.0
Cl2—Cd1—S7	170.53 (6)	C5—C4—H4A	110.0
Cl1^i^—Cd1—S7	94.51 (5)	N2—C4—H4B	110.0
S9—Cd1—S7	84.88 (4)	C5—C4—H4B	110.0
Cl1—Cd1—S7	79.87 (5)	H4A—C4—H4B	108.4
S5—Cd1—S7	90.19 (4)	C4—C5—S6	106.5 (4)
Cd1^ii^—Cl1—Cd1	162.98 (5)	C4—C5—H5A	110.4
C3—S4—C2	92.2 (2)	S6—C5—H5A	110.4
C3—S5—Cd1	108.37 (16)	C4—C5—H5B	110.4
C6—S6—C5	93.7 (3)	S6—C5—H5B	110.4
C6—S7—Cd1	107.80 (17)	H5A—C5—H5B	108.6
C9—S8—C8	93.1 (3)	N2—C6—S7	127.7 (4)
C9—S9—Cd1	109.53 (18)	N2—C6—S6	112.2 (4)
C3—N1—C1	117.3 (4)	S7—C6—S6	120.1 (3)
C3—N1—H1	121.4	N3—C7—C8	107.7 (5)
C1—N1—H1	121.4	N3—C7—H7A	110.2
C6—N2—C4	119.2 (5)	C8—C7—H7A	110.2
C6—N2—H2	120.4	N3—C7—H7B	110.2
C4—N2—H2	120.4	C8—C7—H7B	110.2
C9—N3—C7	118.2 (5)	H7A—C7—H7B	108.5
C9—N3—H3	120.9	C7—C8—S8	106.6 (4)
C7—N3—H3	120.9	C7—C8—H8A	110.4
N1—C1—C2	107.7 (4)	S8—C8—H8A	110.4
N1—C1—H1A	110.2	C7—C8—H8B	110.4
C2—C1—H1A	110.2	S8—C8—H8B	110.4
N1—C1—H1B	110.2	H8A—C8—H8B	108.6
C2—C1—H1B	110.2	N3—C9—S9	127.7 (4)
H1A—C1—H1B	108.5	N3—C9—S8	111.7 (4)
C1—C2—S4	106.1 (4)	S9—C9—S8	120.7 (3)
			
C3—N1—C1—C2	18.9 (6)	Cd1—S7—C6—N2	−32.2 (5)
N1—C1—C2—S4	−21.9 (5)	Cd1—S7—C6—S6	148.6 (2)
C3—S4—C2—C1	17.1 (4)	C5—S6—C6—N2	−1.8 (4)
C1—N1—C3—S5	174.6 (4)	C5—S6—C6—S7	177.5 (3)
C1—N1—C3—S4	−5.7 (5)	C9—N3—C7—C8	−14.1 (7)
Cd1—S5—C3—N1	25.0 (4)	N3—C7—C8—S8	16.8 (6)
Cd1—S5—C3—S4	−154.7 (2)	C9—S8—C8—C7	−13.4 (4)
C2—S4—C3—N1	−7.2 (4)	C7—N3—C9—S9	−175.6 (4)
C2—S4—C3—S5	172.5 (3)	C7—N3—C9—S8	3.8 (6)
C6—N2—C4—C5	3.6 (7)	Cd1—S9—C9—N3	−10.5 (5)
N2—C4—C5—S6	−4.4 (7)	Cd1—S9—C9—S8	170.1 (2)
C6—S6—C5—C4	3.6 (5)	C8—S8—C9—N3	6.1 (4)
C4—N2—C6—S7	−180.0 (4)	C8—S8—C9—S9	−174.4 (3)
C4—N2—C6—S6	−0.8 (6)		

Symmetry codes: (i) −*x*−1, −*y*−1, *z*+1/2; (ii) −*x*−1, −*y*−1, *z*−1/2.

### Hydrogen-bond geometry (Å, º)

**Table:**

*D*—H···*A*	*D*—H	H···*A*	*D*···*A*	*D*—H···*A*
N1—H1···Cl1^i^	0.86	2.43	3.260 (4)	163
N2—H2···Cl1^i^	0.86	2.47	3.314 (4)	168
N3—H3···Cl2	0.86	2.41	3.165 (4)	147
C8—H8*B*···Cl1^iii^	0.97	2.82	3.781 (6)	171

Symmetry codes: (i) −*x*−1, −*y*−1, *z*+1/2; (iii) *x*−1, *y*, *z*.
